# Assessing the relationship between surface urban heat islands and landscape patterns across climatic zones in China

**DOI:** 10.1038/s41598-017-09628-w

**Published:** 2017-08-24

**Authors:** Qiquan Yang, Xin Huang, Jiayi Li

**Affiliations:** 1 School of Remote Sensing and Information Engineering, Wuhan University, 129 Luoyu Road, Wuhan, 430079 China; 2 State Key Laboratory of Information Engineering in Surveying, Mapping and Remote Sensing, Wuhan University, Wuhan, 430079 China

## Abstract

The urban heat island (UHI) effect exerts a great influence on the Earth’s environment and human health and has been the subject of considerable attention. Landscape patterns are among the most important factors relevant to surface UHIs (SUHIs); however, the relationship between SUHIs and landscape patterns is poorly understood over large areas. In this study, the surface UHI intensity (SUHII) is defined as the temperature difference between urban and suburban areas, and the landscape patterns are quantified by the urban-suburban differences in several typical landscape metrics (ΔLMs). Temperature and land-cover classification datasets based on satellite observations were applied to analyze the relationship between SUHII and ΔLMs in 332 cities/city agglomerations distributed in different climatic zones of China. The results indicate that SUHII and its correlations with ΔLMs are profoundly influenced by seasonal, diurnal, and climatic factors. The impacts of different land-cover types on SUHIs are different, and the landscape patterns of the built-up and vegetation (including forest, grassland, and cultivated land) classes have the most significant effects on SUHIs. The results of this study will help us to gain a deeper understanding of the relationship between the SUHI effect and landscape patterns.

## Introduction

Increasingly strong impacts resulting from accelerating urbanization pose a threat to the Earth’s environment^1^. Among these effects, the urban heat island (UHI) effect, i.e., the phenomenon of higher temperatures in urban areas relative to the surrounding areas^2^, has been the subject of considerable attention in recent years^3–8^. The UHI effect has potential influences on energy consumption^9^, vegetation growth^10, 11^, air and water quality^1, 9^, and can even cause harm to human health^9, 12, 13^. Therefore, a better understanding of the UHI effect and its related factors is obviously of critical importance.

UHIs can be broadly divided into atmospheric UHIs calculated from weather station networks, and surface UHIs (SUHIs) evaluated from thermal infrared remote sensing techniques. Compared with the atmospheric UHIs, the SUHIs have the advantages of easier access, wall-to-wall continuous coverage, and direct linkage with surface conditions^5, 14^. Hence, the SUHI effect has been extensively applied for heat island study^3–6, 15–17^.

A number of studies have signified that urban landscape patterns, quantified by landscape metrics^18^ and comprised of two fundamental aspects^19, 20^ (i.e. landscape composition and configuration), are among the most important factors relevant to SUHIs^4, 5, 9, 21, 22^. However, to date, almost all the studies that have investigated the relationship between SUHIs and landscape patterns have been conducted only in a single city^23–30^ or a few cities^21, 31, 32^. The conclusions drawn from these studies may not be comprehensive or may even be mutually contradictory, because of the limitations of the regional climate conditions, geographical locations, development levels, or some other factors of the target city/cities. For instance, some studies have indicated that a higher density of urban development results in a more obvious SUHI effect^21, 26^. However, a quite different result was reported in another investigation, where it was concluded that more sprawling city patterns contribute to the formation of surface heat islands^29^. The conclusion made in some studies that water can significantly alleviate the SUHI phenomenon^28, 32^ has not been supported in other research^26^. Moreover, numerous studies have revealed that SUHIs and the landscape patterns of cities vary obviously with seasonal and diurnal changes^3, 4, 21, 33^. However, most studies that have investigated the relationship between SUHIs and landscape patterns have ignored the seasonal and diurnal factors^24, 26–28^. There is therefore a strong need to understand the correlation between SUHIs and landscape patterns, considering seasonal and diurnal factors, for different cities located in diverse climatic zones.

China has witnessed a rapid urbanization process over the past few decades^34^, and the trend is expected to continue^35^. Sustained and rapid urbanization has not only led to significant UHI effects in many cities of China^3, 4, 22^, but has also profoundly changed China’s urban landscape and land-use patterns^36^. Moreover, China covers diverse climatic conditions, varying from tropical to subarctic/alpine, and changing from arid to humid^37^. Meanwhile, located in the East Asian monsoon region, China experiences obviously seasonal dynamics and environmental changes^38^. These factors make China an ideal study area to investigate the relationship between SUHIs and landscape patterns. Although studies of UHIs and landscape patterns have been carried out in a number of Chinese cities^21, 23, 26, 28^, few studies have systematically quantified the relationship between SUHIs and landscape patterns at the national scale.

**Figure 1:**
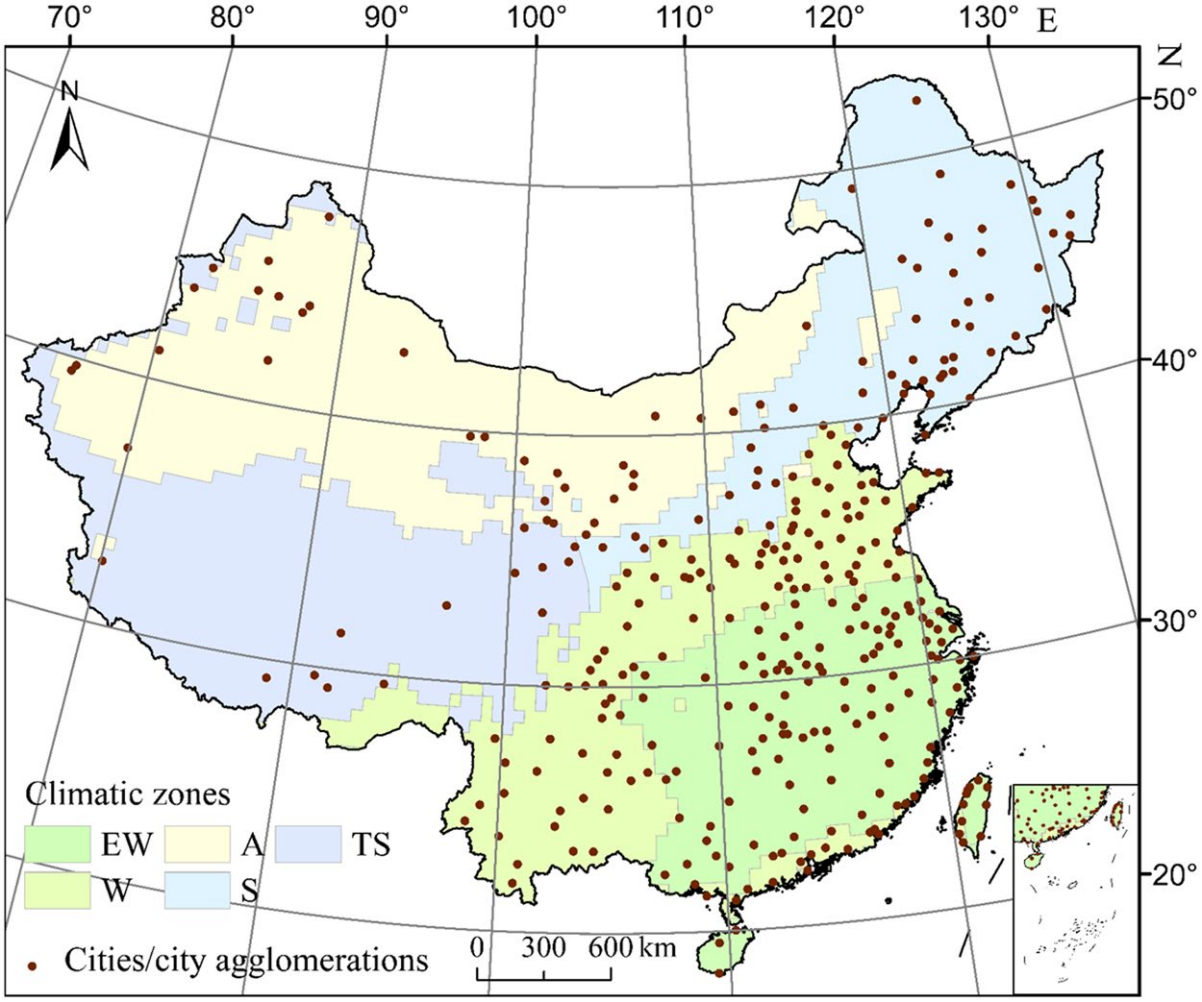
Locations of the 332 cities/city agglomerations and five climatic zones in China. At first, we selected 336 cities in China; however, because of the confusion of urban area borders, several cities (e.g. Jiangmen and Zhongshan in Guangdong province) were aggregated as one city agglomeration and, finally, the 332 cities/city agglomerations shown in the figure were incorporated in the study. Climatic zones were characterized by different climatic conditions according to the Köppen-Geiger climate classification system^48^. EW stands for equatorial climate and warm and fully humid temperate climate, W stands for warm temperate climate with dry winter, A stands for the climate of arid steppe and desert, S stands for snow climate with dry winter, and TS, suited at the Qinghai–Tibet Plateau, stands for tundra climate and snow climate with cool summer and cold winter. This map was generated using ArcGIS 10.0 software (www.esri.com/software/arcgis).

**Figure 2:**
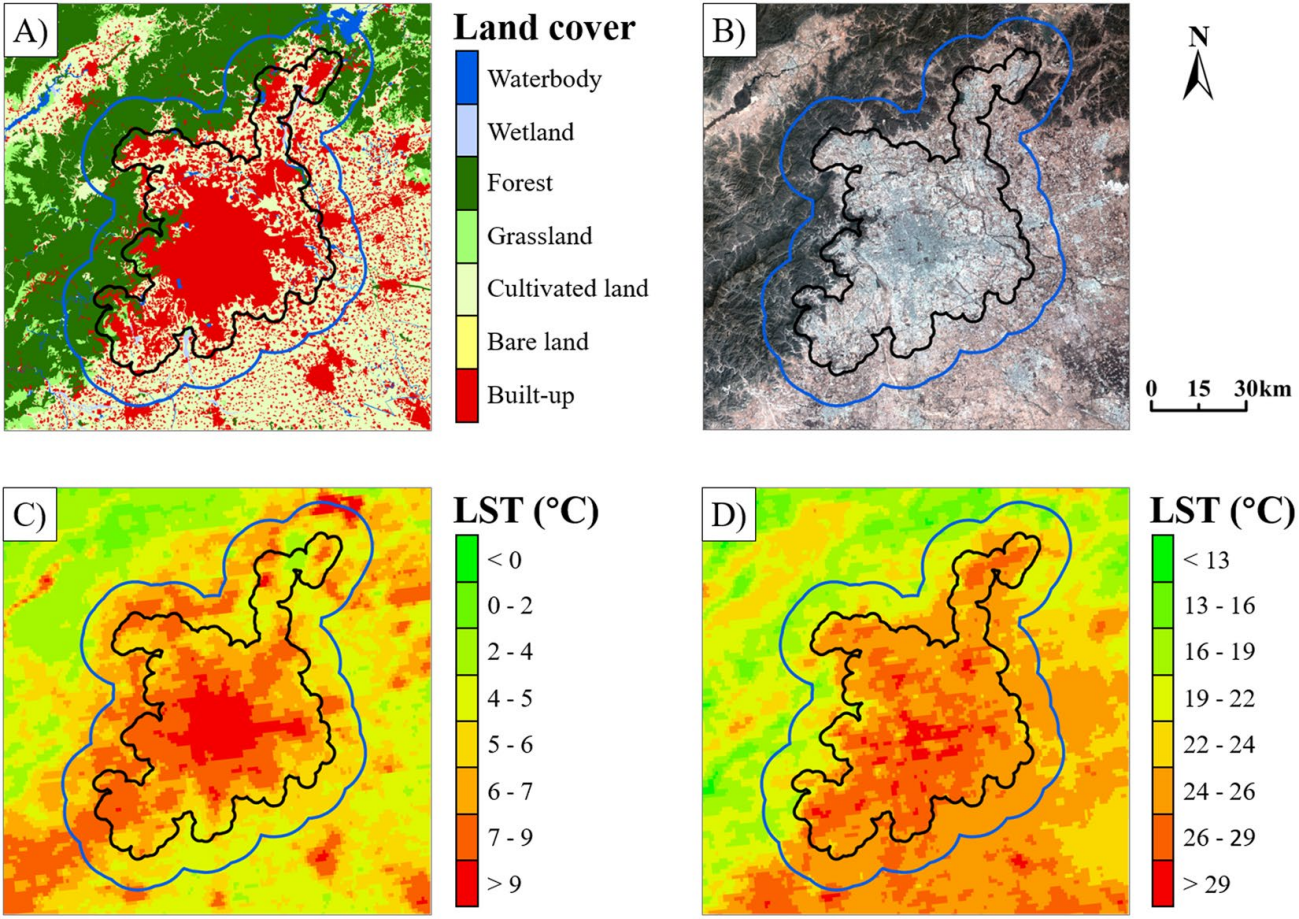
The delineation of urban and suburban areas, using Beijing as an example. (**A**) Land-cover classification from China’s Land-Use/Cover Datasets (CLUDs), with a spatial resolution of 30 m × 3 m. (**B**) Landsat Thematic Mapper true-color image acquired on April 16, 2015. (**C**) Annual mean nighttime land surface temperature (LST, °C). (**D**) Annual mean daytime LST. The black line represents the border of the urban area. The land within the border is considered as the urban area, and that outside the black line but within the blue line represents the suburban area, which covers the same amount of land as the urban area. This map was generated using ArcGIS 10.0 software (www.esri.com/software/arcgis).

The purpose of this study was thus to investigate the SUHI effect and its relationship to the patterns of the urban landscape in 332 cities/city agglomerations distributed in five different climatic zones of China (Fig. 1). Seasonal (i.e. summer and winter) and diurnal (i.e. day and night) factors were considered in the research. The SUHI intensity (SUHII) was defined as the average land surface temperature (LST) difference between an urban area and its surrounding suburban area (Fig. 2), and the landscape patterns were quantified by the urban-suburban differences in several typical landscape metrics (ΔLMs) (Table 1). The Moderate Resolution Imaging Spectroradiometer (MODIS) LST product (version 5) and China’s Land-Use/Cover Datasets (CLUDs) were used to evaluate the SUHII and ΔLMs, respectively (see Methods). The results of this study will help to deepen our understanding of how landscape patterns affect the UHI effect. In addition, new and important insights could be provided to urban planners and managers on how to mitigate the UHI effect from the perspective of urban landscape design, taking into account the climatic conditions of different cities in China.

**Table 1:** Landscape pattern metrics used in the study, after McGarigal *et al*.^54^.

Metric (abbr.)	Calculation and description	Level
**Compositional**		
Percentage of landscape (PLAND)	${\rm{PLAND}}=100\times \sum _{j=\,1}^{n}{a}_{ij}/A$ *n* is the number of patches in the landscape for class *i*; *a*_*ij*_ is the area of patch *ij*; *A* is the total landscape area. It is a measure of the proportion of the total area occupied by a particular land-use type.	Class
Shannon’s diversity index (SHDI)	${\rm{SHDI}}=-\sum _{i=1}^{m}({P}_{i}\times ln{P}_{i})$ *P*_*i*_ is the proportion of the landscape occupied by patch type *i*; SHDI is a measure of the land-cover diversity in the landscape.	Landscape
**Configurational**		
Patch density (PD)	PD = *n*_*i*_/*A n*_*i*_ is the number of patches in the landscape for patch type *i*; *A* is the total landscape area. It is a measure of the density of a particular land-use type in the landscape.	Class, landscape
Mean shape index (MSI)	${\rm{MSI}}=0.25\times \sum _{j=1}^{n}({P}_{ij}/\sqrt{{a}_{ij}})/n$ *n* is the number patches in the landscape for class *i*; *P*_*ij*_ and *a*_*ij*_ are the perimeter and the area of patch *ij*, respectively. MSI increases as the patches become more irregular and complex.	Class, landscape
Clumpiness index (CI)	Given ${G}_{i}=({g}_{ij}/\sum _{i=k}^{m}{g}_{ik})$ $${\rm{CI}}=\{\begin{array}{c}({G}_{i}-{P}_{i})/{P}_{i}\,{P}_{i} > {G}_{i}A{\&}{P}_{i} < 0.5\\ ({G}_{i}-{P}_{i})/(1-{P}_{i})\,else\end{array}$$ *g*_*ij*_ is the number of like adjacencies between pixels of patch type *i* based on the double-count method; *g*_*ik*_ is the number of adjacencies between pixels of patch types *i* and *k* based on the double-count method; *P*_*i*_ is the proportion of the landscape occupied by patch type *i*. The values of CI vary from −1 (maximally disaggregated) to 1 (maximally aggregated), where 0 represents an essentially random distribution.	Class
Contagion index (CONTAG)	${\rm{CONTAG}}=[1+\frac{{\sum }_{i=1}^{m}{\sum }_{k=1}^{m}[({P}_{i})(\frac{{g}_{ik}}{{\sum }_{k=1}^{m}{g}_{ik}})]\times [ln({P}_{i})(\frac{{g}_{ik}}{{\sum }_{k=1}^{m}{g}_{ik}})]}{2ln(m)}]100$ *P*_*i*_ is the proportion of the landscape occupied by patch type *i*; *g*_*ik*_ is the number of adjacencies between pixels of patch types *i* and *k* based on the double-count method; *m* is the number of patch types present in the landscape. CONTAG describes the aggregation of all the patch types.	Landscape

## Results

### SUHIs and landscape patterns across climatic zones

The SUHI intensities (i.e. SUHII) of 332 Chinese cities/city agglomerations were calculated in this study, and the average SUHII values across five climatic zones and China are shown in Table 2. In the winter daytime, the average SUHII of the EW climatic zone (equatorial climate and warm and fully humid temperate climate), situated in the southeast of China, is clearly higher than that of any other climatic zone. Meanwhile, in the winter nighttime, the average SUHII in the EW climatic zone is the lowest, and the most intense average SUHII occurs in climatic zone A (arid steppe and desert climate), which is mainly located in the northwest of China. Interestingly, in the summer daytime, climatic zone S (snow climate with dry winter) and climatic zone TS (tundra climate and snow climate with cool summer and cold winter) experience the highest and lowest SUHII, respectively. Seasonally, the SUHII in summer is mostly higher than that in winter. Diurnally, the daytime SUHII is higher than the nighttime SUHII in summer, but the opposite phenomenon occurs in winter (except for climatic zone EW).

**Table 2:** Annual, summer, and winter daytime and nighttime surface urban heat island intensity (SUHII, °C, Mean ± SD) across climatic zones (EW, W, A, S and TS) and China.

*N*	EW	W	A	S	TS	China
	121	109	31	56	15	332
Annual daytime	1.11 ± 0.46	1.24 ± 0.71	0.80 ± 1.15	1.44 ± 1.23	0.58 ± 1.01	1.16 ± 0.84
Annual nighttime	0.47 ± 0.26	0.79 ± 0.46	0.96 ± 0.50	0.78 ± 0.55	0.61 ± 0.54	0.68 ± 0.46
Summer daytime	1.91 ± 0.79	1.78 ± 0.86	1.16 ± 1.29	1.97 ± 0.96	0.93 ± 1.02	1.76 ± 0.95
Summer nighttime	0.54 ± 0.25	0.84 ± 0.47	1.01 ± 0.46	0.75 ± 0.41	0.68 ± 0.62	0.73 ± 0.43
Winter daytime	0.77 ± 0.59	0.45 ± 0.80	0.22 ± 0.92	0.41 ± 0.81	0.28 ± 1.00	0.53 ± 0.77
Winter nighttime	0.39 ± 0.31	0.71 ± 0.51	0.95 ± 0.51	0.87 ± 0.78	0.51 ± 0.53	0.63 ± 0.55

See Fig. 1 for details of the climatic zones. Summer and winter are defined as the periods from June to August, and from December to February, respectively. *N* indicates the number of cities/city agglomerations in each climatic zone and China.

The landscape metrics applied in this study were: (1) percentage of landscape (PLAND); (2) Shannon’s diversity index (SHDI); (3) patch density (PD); (4) mean shape index (MSI); (5) clumpiness index (CI); and (6) contagion index (CONTAG). These landscape metrics were calculated at both the class and landscape levels. Class-level metrics describe the characteristics of each single type of land cover, while landscape-level metrics examine the spatial structure in multi-class patch mosaics (see Methods and Table 1 for more details). At the landscape level, ΔPD (“Δ” means the difference between urban and suburban) and ΔCONTAG are both negative, while ΔSHDI and ΔMSI are both positive in nearly all the climatic zones (Supplementary Table S1). At the class level, the signs of the ΔLMs depend on both the climatic zones and the land-cover types. For instance, the ΔPD values are all no more than zero, while ΔPLAND is negative in almost all the climatic zones for every land-cover type, except for the built-up class (Supplementary Table S1). On average, the percentages of built-up and cultivated land are both above 30% for the urban areas in nearly all the climatic zones (except for the climatic zone situated at the Qinghai–Tibet Plateau (TS)), generally followed by forest, grassland, bare land, wetland, and waterbody (Supplementary Table S2).

### The relationship between SUHIs and landscape patterns

Spearman’s rank correlation coefficients of the SUHII and ΔLMs across climatic zones and China were applied to quantify the relationship between SUHIs and landscape patterns. At the class level, we investigated the correlations between SUHIs and landscape patterns for seven different types of land cover (see Methods), respectively.

For the built-up class, SUHII is significantly (p < 0.05) and positively correlated with ΔPLAND for most cases (Table 3). In contrast, ΔCI is mostly negatively correlated with SUHII. It should be noted that, in the winter daytime, the correlation between SUHII and ΔPD is significantly positive in the EW (r = 0.40, p < 0.001) and W (r = 0.58, p < 0.001) climatic zones, but is not significant in the A (r = 0.18, p > 0.05), S (r = 0.08, p > 0.05), and TS (r = −0.02, p > 0.05) climatic zones. Similarly, the winter daytime SUHII has a significant and positive correlation with ΔMSI in the EW (r = 0.24, p < 0.01) and W (r = 0.39, p < 0.001) climatic zones, but it is not significantly correlated with ΔMSI in the other climatic zones.

**Table 3:** Spearman’s rank correlation coefficients between the surface urban heat island intensity (SUHII) and the urban-suburban difference in the landscape metrics (ΔLMs) of the built-up class across climatic zones and China.

Landscape metrics for the built-up class	Climatic zone	Annual day	Annual night	Summer day	Summer night	Winter day	Winter night
ΔPLAND	China	0.27^a^	0.40^a^	0.32^a^	0.37^a^	−0.04	0.43^a^
	EW	0.19^c^	0.14	0.26^b^	0.18^c^	−0.02	0.25^b^
	W	0.20^c^	0.46^a^	0.27^b^	0.31^b^	−0.15	0.45^a^
	A	0.43^c^	0.56^a^	0.20	0.66^a^	0.20	0.41^c^
	S	0.33^c^	0.59^a^	0.42^b^	0.62^a^	0.01	0.56^a^
	TS	0.06	0.38	0.20	0.50	−0.26	0.41
ΔPD	China	−0.10	−0.27^a^	−0.14^b^	−0.07	0.31^a^	−0.31^a^
	EW	0.07	0.07	−0.07	0.31^a^	0.40^a^	−0.08
	W	0.01	−0.56^a^	0.14	−0.25^b^	0.58^a^	−0.63^a^
	A	−0.34	−0.26	−0.73^a^	−0.20	0.18	−0.17
	S	−0.30^c^	−0.43^b^	−0.38^b^	−0.40^b^	0.08	−0.40^b^
	TS	−0.05	0.29	−0.17	0.10	−0.02	0.40
ΔMSI	China	−0.07	−0.25^a^	−0.15^b^	−0.11^c^	0.23 ^a^	−0.27^a^
	EW	−0.05	0.20^c^	−0.17	0.14	0.24^b^	0.10
	W	0.02	−0.44^a^	0.06	−0.16	0.39^a^	−0.48^a^
	A	0.09	−0.10	−0.27	0.00	0.34	−0.21
	S	−0.23	−0.53^a^	−0.36^b^	−0.46^a^	−0.02	−0.50^a^
	TS	0.14	0.06	−0.14	0.03	0.33	0.23
ΔCI	China	−0.18^a^	−0.13^c^	−0.02	−0.11^c^	−0.11^c^	−0.12^c^
	EW	−0.22^c^	−0.07	−0.01	0.00	−0.31^a^	−0.05
	W	−0.14	−0.06	−0.05	−0.03	−0.13	−0.03
	A	−0.28	−0.06	−0.06	−0.17	−0.21	−0.05
	S	0.12	−0.19	0.17	−0.10	0.20	−0.12
	TS	−0.32	−0.26	−0.54^c^	−0.23	0.04	−0.14

See Fig. 1 and Table 1 for details of the climatic zones (EW, W, A, S, and TS) and landscape metrics (PLAND, PD, MSI, and CI), respectively. Δmeans the difference between urban and suburban. ^a^Significant at the 0.001 level; ^b^significant at the 0.01 level; ^c^significant at the 0.05 level.

The correlations between SUHII and the ΔLMs, in most circumstances, are not significant for bare land, waterbody, and wetland (Supplementary Tables S3–S5). At the national scale, for bare land, ΔPLAND is generally positively correlated with the daytime SUHII (r = 0.14, p < 0.05 for annual; r = 0.19, p < 0.05 for summer; r = 0.06, p > 0.05 for winter), and ΔPLAND shows a weak-and-negative correlation with the nighttime SUHII (r = −0.18, p < 0.05 for annual; r = −0.18, p < 0.05 for summer; r = −0.20, p < 0.05 for winter). In contrast, for waterbody and wetland, ΔPLAND is generally negatively correlated with daytime SUHII and positively correlated with nighttime SUHII across China.

Although the forest, grassland, and cultivated land classes all belong to vegetation, the relationships between their landscape patterns and SUHIs appear quite different when seasonal, diurnal, and climatic factors are taken into consideration (see Table 4 and Supplementary Tables S6–S8). For the forest class, the daytime SUHII usually shows negative correlations with ΔPLAND, and most of these negative correlations are significant in summer (Table 4). In comparison, the nighttime SUHII usually shows positive correlations with the ΔPLAND of forest (except for the EW climatic zone), but the majority of these positive correlations are not significant (Table 4). From summer to winter, the daytime alleviation effect of ΔPLAND becomes weaker in the north of China (e.g. for climatic zone A, r = −0.46, p < 0.05 in summer daytime, and r = −0.05, p > 0.05 in winter daytime), and the nighttime enhancement effect of ΔPLAND becomes stronger in many climatic zones (e.g. for climatic zone W, r = 0.01, p > 0.05 in summer nighttime, and r = 0.48, p < 0.001 in winter nighttime) (Table 4). Specifically, the ΔPD of forest is mostly negatively correlated with daytime SUHII and positively correlated with nighttime SUHII (Supplementary Table S6). In addition, there is no significant correlation between SUHII and ΔMSI or ΔCI, in most cases (Supplementary Table S6).

**Table 4:** Spearman’s rank correlation coefficients between the surface urban heat island intensity (SUHII) and the urban-suburban difference in the percentage of landscape (ΔPLAND) of vegetation (including forest, grassland, and cultivated land) across five climatic zones and China.

ΔPLAND of vegetation	Climatic zone	Annual day	Annual night	Summer day	Summer night	Winter day	Winter night
ΔPLAND of forest	China	−0.22^a^	0.18^a^	−0.27^a^	−0.03	−0.47^a^	0.27^a^
	EW	−0.12	−0.24^b^	−0.04	−0.54^a^	−0.50^a^	−0.07
	W	−0.36^a^	0.30^b^	−0.43^a^	0.01	−0.68^a^	0.48^a^
	A	−0.16	0.07	−0.46^c^	0.02	−0.05	0.01
	S	−0.12	0.17	−0.26^c^	−0.12	−0.07	0.21
	TS	−0.15	0.60^c^	0.10	0.49	−0.15	0.57^c^
ΔPLAND of grassland	China	0.09	−0.09	0.16^c^	−0.18^b^	0.03	−0.02
	EW	−0.16	−0.05	−0.14	−0.18	−0.31^a^	0.15
	W	−0.08	0.03	−0.07	−0.11	−0.13	0.14
	A	0.10	0.01	0.42^c^	−0.05	−0.02	0.00
	S	0.29^c^	0.07	0.44^b^	−0.02	0.04	0.13
	TS	0.19	−0.66^b^	−0.10	−0.64^c^	0.41	−0.71^b^
ΔPLAND of cultivated land	China	−0.07	−0.31^a^	−0.11^c^	−0.08	0.38^a^	−0.42^a^
	EW	0.12	0.14	−0.05	0.39^a^	0.60^a^	−0.13
	W	0.14	−0.50^a^	0.15	−0.16	0.58^a^	−0.63^a^
	A	−0.61^a^	−0.40^c^	−0.76^a^	−0.40^c^	−0.11	−0.26
	S	−0.32^c^	−0.63^a^	−0.39^b^	−0.39^b^	0.05	−0.64^a^
	TS	0.25	0.21	0.28	0.26	0.11	0.25

The results of the other landscape metrics (i.e. PD, MSI, and CI) are shown in Supplementary Tables S6–S8. See Fig. 1 and Table 1 for details of the climatic zones (EW, W, A, S, and TS) and landscape metrics (PLAND, PD, MSI, and CI), respectively. Δ means the difference between urban and suburban. ^a^Significant at the 0.001 level; ^b^significant at the 0.01 level; ^c^significant at the 0.05 level.

For the grassland class, the increase of ΔPLAND generally has no significant mitigating effect on SUHII in the daytime, and ΔPLAND is even significantly and positively correlated with summer daytime SUHII in the A (r = 0.42, p < 0.05) and S (r = 0.44, p < 0.05) climatic zones (Table 4). Similar to the forest class, SUHII is not significantly correlated with the ΔMSI or ΔCI of grassland (Supplementary Table S7). However, it should be noted that, in the TS climatic zone, SUHII is significantly negatively correlated with ΔPLAND in the nighttime and ΔPD in the daytime, respectively (Table 4 and Supplementary Table S7).

For the cultivated land class, ΔPLAND is significantly correlated with SUHII in nearly all the climatic zones (except for climatic zone TS) (Table 4). In summer, the correlations between SUHII and ΔPLAND are significantly negative in the A and S climatic zones, but are rarely significant in the EW or W climatic zones. In winter daytime, SUHII is significantly and positively correlated with ΔPLAND in the EW and W climatic zones. In addition, the relationship between the ΔCI of cultivated land and SUHII is negative in winter daytime, but positive in winter nighttime (except for climatic zone TS), and most of the correlations between ΔMSI and SUHII are not significant (Supplementary Table S8).

At the landscape level, SUHII is generally positively correlated with ΔCI and negatively correlated with ΔSHDI in all the climatic zones, except for TS (where the opposite correlation occurs). Furthermore, there is no significant relationship between SUHII and ΔPD or ΔMSI, in most cases (Table 5).

**Table 5:** Spearman’s rank correlation coefficients between the surface urban heat island intensity (SUHII) and the urban-suburban difference in the metrics at the landscape level across five climatic zones and China.

Metrics at the landscape level	Climatic zone	Annual day	Annual night	Summer day	Summer night	Winter day	Winter night
ΔPD	China	−0.08	−0.03	−0.09	−0.02	0.12^c^	−0.03
	EW	0.01	0.10	−0.04	0.18	0.08	−0.04
	W	−0.04	−0.05	−0.01	−0.05	0.16	0.00
	A	−0.10	0.27	−0.45^c^	0.23	0.08	0.26
	S	−0.05	−0.03	−0.09	−0.25	0.11	0.04
	TS	−0.19	0.58^c^	−0.16	0.44	−0.31	0.66^b^
ΔMSI	China	0.15^b^	−0.04	0.02	−0.02	0.06	−0.09
	EW	0.23^c^	−0.10	0.09	−0.14	0.08	−0.14
	W	0.15	−0.10	0.07	0.03	0.17	−0.17
	A	0.02	−0.33	−0.17	−0.25	0.19	−0.48^b^
	S	0.09	−0.20	0.04	−0.27^c^	0.11	−0.16
	TS	0.14	−0.06	0.24	−0.06	0.15	−0.05
ΔCONTAG	China	0.16^b^	0.13^c^	0.17^b^	0.13^c^	0.06	0.08
	EW	0.15	0.06	0.08	−0.02	0.20^c^	0.01
	W	0.15	0.16	0.16	0.15	0.06	0.07
	A	0.23	0.09	0.35	0.11	−0.01	0.12
	S	0.10	0.34^c^	0.12	0.46^a^	−0.07	0.23
	TS	−0.06	−0.53^c^	−0.16	−0.45	0.12	−0.59^c^
ΔSHDI	China	−0.18^a^	−0.14^c^	−0.17^b^	−0.14^c^	−0.05	−0.09
	EW	−0.17	−0.08	−0.10	−0.03	−0.26^b^	−0.01
	W	−0.16	−0.16	−0.19^c^	−0.16	−0.04	−0.06
	A	−0.31	−0.04	−0.46^c^	−0.11	−0.04	−0.02
	S	−0.11	−0.32^c^	−0.12	−0.46^a^	0.08	−0.26
	TS	0.11	0.76^c^	0.28	0.73^b^	−0.13	0.82^c^

See Fig. 1 and Table 1 for details of the climatic zones (EW, W, A, S, and TS) and landscape metrics (PD, MSI, CONTAG, and SHDI), respectively. Δ means the difference between urban and suburban. ^a^Significant at the 0.001 level; ^b^significant at the 0.01 level; ^c^significant at the 0.05 level.

## Discussion

### The necessity of taking seasonal, diurnal, and climatic factors into account

**Figure 3:**
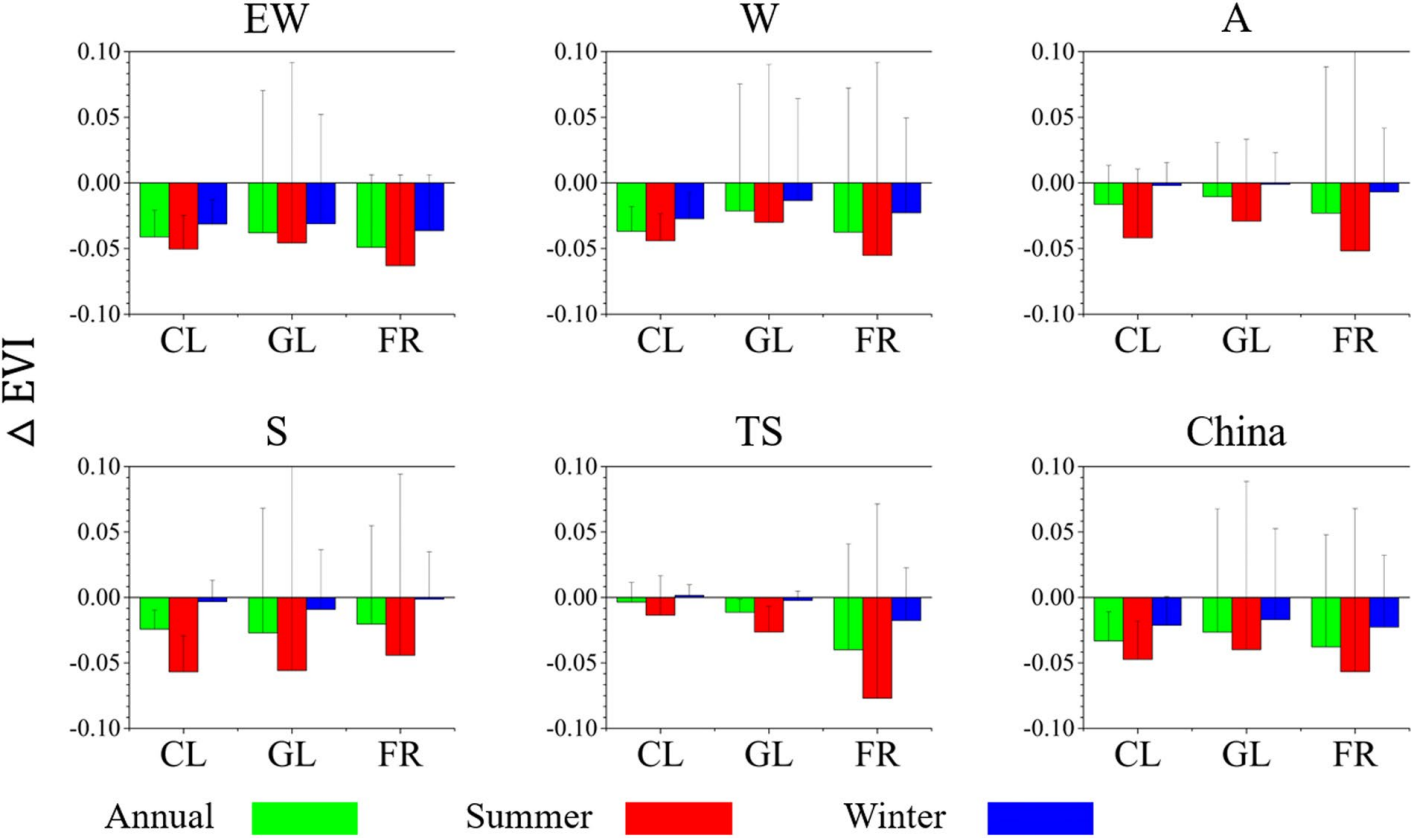
The urban-suburban difference (mean + SD) in the Enhanced Vegetation Index (ΔEVI) of different types of vegetation (including cultivated land (CL), grassland (GL), and forest (FR)) across climatic zones and China (annual, summer, and winter). See Fig. 1 for details of the climatic zones (EW, W, A, S, and TS). This map was generated using Origin 9.0 software (http://www.originlab.com/).

The spatio-temporal heterogeneity of the UHI effect in China is explicitly delineated in this study (Table 2). Firstly, Seasonal differences of SUHII are observed in this study, which might be relevant to the change of the physical and biochemical properties of the land cover^3, 4^. For instance, through calculating the urban-suburban differences in the Enhanced Vegetation Index (ΔEVI) of the different vegetation types, we can clearly see the seasonal variation of vegetation (Fig. 3). Secondly, we found obvious differences between daytime and nighttime SUHII (Table 2), and these diurnal differences are probably attributed to the fact that the mechanism of daytime heat island formation is different from that at night^3, 4, 17, 22^. The daytime SUHII was widely considered to be the result of an increase in sensible heat flux and a reduction in latent heat flux due to large areas of vegetated and evaporating soil surfaces are encroached by impervious surface^2–4, 39^, while the release of the more stored energy in the urban zone compared to surrounding area contributes to nighttime heat island^4, 17^. Thirdly, the intensities of the SUHIs in different climatic zones usually show differences (Table 2). Similar to the findings in a previous investigation^4^, the southeast of China (EW) tends to experience stronger SUHII in the daytime and weaker SUHII in the nighttime. Many cities located in the northwest of China (A) and the Qinghai–Tibet Plateau (TS) were included in this study, and the average SUHII in the two climatic zones (i.e. A and TS) also shows unique characteristics. Finally, from the perspective of landscape patterns, the values of the landscape metrics in the urban areas and the urban-suburban difference of the landscape metrics (i.e. ΔLMs) both vary with the change of land-cover types and climatic zones (Supplementary Tables S1 and S2). Therefore, it is important to take seasonal, diurnal, and climatic factors into account when investigating the quantitative correlations between SUHIs and landscape patterns.

### Theoretical implications of the relationship between SUHIs and landscape patterns

The results of Spearman’s rank correlation analysis show that the relationships between ΔLMs of different land-cover types and SUHII are different, and these relationships are closely related with seasonal, diurnal and climatic factors (Tables 3–5, and Supplementary Tables S3–S8). Generally speaking, three reasons account for this. Firstly, the SUHII itself is related with seasonal, diurnal and climatic factors, and ΔLMs also vary with climatic regions and land-cover types. Accordingly, from a statistical point of view, the relationships between SUHIs and landscape patterns shall be likely connected with seasonal, diurnal and climatic factors. Secondly, different land-cover types cause different feedbacks to LST and further SUHII, due to the variation of their physical and biochemical properties^21, 31, 40^. For instance, the results of Wang *et al*. showed the variation and differences of albedo, surface roughness and aerodynamic resistance across the cultivated land, built-up and grassland^31^. Thirdly, local climatic conditions, including precipitation, temperature, soil moisture and air humidity, have notable influences on the correlation between urban landscapes and heat island effects^41, 42^. The detailed discussions of the relationships between SUHII and ΔLMs of each type of land cover are given separately below.

The built-up class, as one of the most important land-cover types in urban areas, can be expected to have a great effect on SUHII. Significantly positive correlations between ΔPLAND of the built-up class and SUHII were observed in this study (Table 3), which indicates that the larger percentage difference of the built-up class between urban and suburban areas has the potential to result in more intense SUHII in the cities. This can be directly explained by the physical characteristics of the built-up class. Built-up areas tend to have lower albedo, higher thermal conductivity, and larger heat capacity, which will result in higher surface heat storage^3, 28, 43^. Besides, with the increase of built-up areas, the heat loss ability in urban areas will be weaker due to denser and taller buildings^21^. Furthermore, some other SUHI-induced factors, including population, energy consuming and air pollution, are closely relevant to the percent of built-up class^43–45^. These factors will indirectly enhance SUHI effects as the ΔPLAND of the built-up class increases. Several studies, focusing on big cities in China such as Beijing, Shanghai, and Wuhan, have reported that the increase of the patch density, aggregation, and irregularity of the built-up class results in higher LST^25, 26, 28^. However, the results in this study indicate that the relationships between SUHII and the characteristics of the built-up class vary with the climatic zones. For instance, the correlations between the winter daytime SUHII and the urban-suburban difference in patch density (i.e. ΔPD) are significantly positive in the EW and W climatic zones, but are not significant and even weakly negative across climatic zones A and S (Table 3). The following two reasons may contribute to this. Firstly, the average patch density of the built-up class in the urban areas of cities located in the EW and W climatic zones is higher than that of the cities located in the A and S climatic zones (Supplementary Table S2). Thus, the built-up patches in the urban area of a city located in climatic zones EW or W connect more easily with each other than those in the urban area of a city located in climatic zones A or S when the patch density of the built-up class in the urban area keeps growing. A previous study concluded that the contiguity of urban buildings, regardless of their density, was the critical factor influencing the magnitude of the UHI effect^46^. Therefore, a positive relationship between SUHII and ΔPD for the built-up class occurs in the EW and W climatic zones, rather than the A and S climatic zones. Secondly, the difference of geographic locations may be another possible reason for the different characteristics of the correlations between SUHII and ΔPD for the built-up class in these two groups of climatic zones. Climatic zones A and S are located in the north of China and have a higher latitude than climatic zones EW and W. As a result, less solar radiation shall be received because of the shorter hours of sunlight and lower solar altitude in winter daytime. Furthermore, in winter daytime, shadows are more easily formed by buildings in climatic zones A and S due to their lower solar altitude^21^, which reduces the amount of shortwave radiation, and thus decreases the LST in urban areas.

Because of the high thermal capacity and inertia of water^21^, the wetland and waterbody classes are able to maintain a low temperature in the day, but a relatively high temperature in the night. Therefore, the increase of the urban-suburban difference in the percentage (ΔPLAND) of wetland and waterbody results in a weaker SUHII in the daytime (13:30 pm), but a more intense SUHII in the nighttime (01:30 am) (Supplementary Tables S4 and S5). In contrast, the ΔPLAND of the bare land class is positively correlated with daytime SUHII, but is negatively correlated nighttime SUHII (Supplementary Table S3). The lower water retention rate of bare land is a possible reason for this opposite correlation. However, most of the correlations between SUHII and the ΔLMs of wetland, waterbody, and bare land are not significant (Supplementary Tables S3–S5), which might be due to their very low percentages (1.88 ± 2.66% for wetland, 0.04 ± 0.05% for waterbody, and 3.76 ± 10.9% for bare land in urban areas at the national level, Supplementary Table S2).

Vegetation has a particular functionality for the increase of the latent heat flux and the decrease of the sensible heat flux via transpiration. It can therefore be expected to have a cooling effect on LST, and thus relieve the SUHI effect^3–5^. However, our results indicate that the relationships between landscape patterns of vegetation and SUHIs are very complex, which need in-depth and detailed analyses.

In summer daytime, the increase of the percentage difference of forest between urban and suburban areas (i.e. the ΔPLAND of forest) significantly mitigates the SUHII in many climatic zones except for EW and TS (Table 4). In climatic zone EW, the insignificant mitigation effect of the ΔPLAND of forest is probably due to the aerodynamically smoother in urban areas than surrounding suburban areas^31, 41^. The cities in climatic zone EW are suited at the southeast of China and have a humid and warm climate in summer. Accordingly, the increase of the ΔPLAND of forest should seem to be able to significantly mitigate the SUHII in EW climatic zone, because the denser vegetation in humid climatic regions has a higher evaporation rate than that in dry climatic regions^41, 47^. However, at the same time, urban areas will have higher aerodynamically resistance to sensible heat diffusion due to the denser vegetation (especially forest) in surrounding suburban areas^41^. As a result, the convection of dissipating heat from urban areas is less efficient than from the surrounding suburban areas in cities located in the EW climatic zone, which will offset evaporation cooling effect to a great degree. This may be the reason of the insignificant effect of the ΔPLAND of forest in the EW climatic zone. In comparison, the insignificant correlation between SUHII and the ΔPLAND of forest in the TS climatic zone (Table 4) is possibly due to the particular climatic characteristics of the Qinghai–Tibet Plateau. In winter daytime, SUHII is significantly and negatively correlated with the ΔPLAND of forest in the EW and W climatic zones, but is insignificantly correlated in the other climatic zones (Table 4). This is primarily due to the lower ΔEVI of forest in climatic zones A, S, and TS than in climatic zones EW and W (Fig. 3). However, the ΔPLAND of forest is insignificantly correlated with summer nighttime SUHII in nearly all the climatic zones (except EW), and is positively correlated with winter nighttime SUHII in the W and TS climatic zones (Table 4), which can likely be attributed to the absence of transpiration in the nighttime^3, 4^.

Grassland, another important type of vegetation, cannot significantly relieve the SUHI effect in most cases, and even enhances the summer daytime SUHII effect in the A and S climatic zones (Table 4). There are two possible reasons for this. Firstly, compared with forest, grassland generally maintains lower rates of evapotranspiration, and thus has a weaker mitigating effect on SUHII^47^. Secondly, the percentage of grassland is usually less than other types of vegetation (13.57 ± 15.27% for forest, 38.62 ± 14.27% for cultivated land, and only 8.58 ± 13.38% for grassland in urban areas at the national scale, Supplementary Table S2). However, the percentage of grassland in the TS climatic zone is relatively high (36.41 ± 25.12%), and several ΔLMs of Grassland are significantly and negatively correlated with SUHII in the TS climatic zone (Table 4 and Supplementary Table S7).

The effect of cultivated land on SUHII cannot be ignored, because of its relatively high proportion in urban areas across China (except for TS) (Supplementary Table S2). In summer, the increase of the ΔPLAND of cultivated land helps to mitigate SUHII in the A and S climatic zones, but it enhances nighttime SUHII in the EW and W climatic zones (Table 4). The uneven distribution of the two main types of cultivated land, i.e., most of the paddy fields are found in south and central China and the dry farming land is mainly located in the north of China^38^, might be able to explain this difference. From summer daytime to winter daytime, the mitigating effect of ΔPLAND on SUHII weakens and even turns into an enhancing effect (Table 4), which is probably due to harvesting resulting in a land-cover change from plants to bare land^31^.

At the landscape level, both the proportion and distribution of the different land-cover types have a pronounced effect on SUHII (Table 5). We found that the increase of the urban-suburban difference in SHDI and CONTAG decrease and increase SUHII, respectively, which is consistent with previous findings^26, 28^. This suggests that more diverse land-use types and more homogeneous mixing of them in the urban area could help to relieve the SUHI effect, which is important information for urban planning. For instance, in order to reduce the urban surface temperature, we could increase the degree of mutual mixing between buildings and vegetation^25^.

### Significance and uncertainties

In contrast to previous studies on the relationship between the UHI effect and landscape characteristics, which have generally been limited to a single city or a small number of cities, we extended the study area to a national scale, with 332 cities/city agglomerations distributed in various climatic zones. In addition, seasonal and diurnal factors were also taken into consideration, and both class and landscape levels were considered in the study. Our research and findings can therefore be considered to be more comprehensive. For instance, the conclusion that the patch density of the built-up class can significantly enhance UHI intensity has been supported in many studies confined to certain big cities^25, 26, 28^. However, our study found that it is only in regions with a relatively high building density that the increase of the patch density of the built-up class in the urban area significantly increases SUHII, but not in regions with a low building density. Furthermore, we found that although forest, grassland, and cultivated land classes belong to vegetation, the impacts of their landscape patterns on SUHII show different characteristics, which has been not revealed in other studies^3, 4^. Overall, the results of this study will help us to gain deeper insights into the relationship between the SUHI effect and landscape patterns.

Some possible uncertainties remain in this research. Firstly, because of the enormous temperature variation of cities in the different regions of China, the direct assessment of the relationship between LST and landscape patterns was not possible in this research. Therefore, in this study, we adopted another way of analyzing the correlation between the urban-suburban difference in surface temperature (i.e. SUHII) and the corresponding difference in landscape patterns (i.e. ΔLMs). Therefore, the difference in the analysis methods needs to be considered when conducting a comparative discussion. Secondly, the numbers of cities in the different climatic zones are different, which is due to the difference in the city densities resulting from the regionally unbalanced development in China. Thirdly, we found that the relationship between SUHII and the ΔLMs in the TS climatic zone, located in the Qinghai–Tibet Plateau, is dissimilar to those in the other climatic zones. The particular geographic location, the special surface conditions, and the lower level of urban development of cities in the Qinghai–Tibet Plateau may be possible reasons for this phenomenon, but the actual causes of this result need to be further investigated.

## Methods

### Study areas

We divided China into five climatic zones (Fig. 1) characterized by different climatic conditions (i.e. temperature and precipitation) according to the Köppen-Geiger climate classification system^48^. In total, 336 cities are distributed in the different climatic zones, including 305 prefecture-level cities, 23 autonomous prefectures, and four municipalities (i.e. Beijing, Shanghai, Tianjin, and Chongqing). Several cities (e.g. Jiangmen and Zhongshan in Guangdong province) were aggregated as one city agglomeration due to the urban areas of these cities being spatially contiguous as a city-cluster. Finally, the analysis addressed 332 cities/city agglomerations (Fig. 1).

### Datasets

The 2015 China Land-Use/Cover Datasets (CLUDs) with a resolution of 30 m were applied to extract the cities’ urban areas and calculate the landscape metrics. The CLUDs were provided by the Chinese Academy of Sciences, and the overall accuracy of the 25 categories of these datasets has been reported as being as high as 90%^49, 50^. We reclassified the categories into eight classes: bare land, built-up, cultivated land, grassland, forest, waterbody, wetland, and permanent snow and ice. Due to the inexistence of permanent snow and ice in most of the cities, only the first seven classes were taken into consideration when computing the landscape metrics. LST was obtained from the EOS-Aqua-MODIS 8-day composite product (version 5) with a spatial resolution of 1 km (MYD11A2) during the period from 2014 to 2016. The Aqua MODIS LST data were acquired in both the daytime (13:30 pm) and nighttime (01:30 am), using a split-window algorithm^51^. The retrieval of LST was further improved by correcting the noise from cloud contamination, topographic differences, and zenith angle changes, obtaining an accuracy of better than 1 K^51, 52^. Considering the seasonal and diurnal factors, we calculated the daytime and nighttime average LST both annually and for summer (from June to August) and winter (from December to February). The Shuttle Radar Topography Mission (SRTM) 3 arc-second (approximately 90 m) digital elevation model (downloaded from http://earthexplorer.usgs.gov/) was also utilized in order to exclude the altitude effect. The MODIS EVI products are able to reflect the spatial distribution and seasonal variation of vegetation. Seasonally average EVI data (MYD13A3) from 2014 to 2016 were employed to estimate the difference of vegetation between urban and suburban areas.

### Landscape metrics

Numerous landscape metrics have been developed to characterize landscape patterns, for both composition and configuration^18, 19^. For this study, we selected six landscape metrics (Table 1) according to the following principles^28, 36, 53^: (1) commonly used; (2) minimal redundancy; and (3) interpretable. The landscape composition was characterized by PLAND and SHDI. PLAND is the most frequently applied composition metric^25^, and SHDI, a popular measurement of a landscape’s diversity, increases as the number of different patch types increases. PD, MSI, CI, and CONTAG were used to describe the landscape configurational features^54^. PD is equal to the number of patches of the corresponding patch type divided by the entire landscape area, and a high PD usually signifies a more fragmented landscape. MSI is an effective indicator to characterize the complexity of landscapes, and a high MSI is generally due to the more irregular and complex shape of patches. CI and CONTAG are both aggregation metrics, and more aggregated landscapes usually correspond to higher values of these metrics. These landscape metrics were computed with the FRAGSTATS public domain software^54^. SHDI and CONTAG were calculated only at the landscape level, CI was computed only at the class level, and the other metrics were calculated at both levels. Considering the definition of SUHII, we calculated the average value differences of all the landscape metrics between urban and suburban areas (i.e. ΔLMs), and our main objective was to comprehensively and systematically analyze the relationship between SUHII and the ΔLMs.

### Analysis

In this study, we defined SUHII as the LST difference between an urban area and its surrounding suburban area^3, 4, 21^. The delineation of urban and suburban areas was based on the method proposed by Zhou *et al*.^4^, according to the land-cover map (i.e. CLUDs). The suburban area (excluding water pixels) within a ring zone around the urban area covered the same area as the urban area (excluding water pixels) (Fig. 2). In order to reduce bias, suburban pixels that satisfied one of the following two conditions were excluded when calculating SUHII: (1) suburban pixels with elevations more than 50 m higher than the highest point or more than 50 m lower than the lowest point in the urban area^5, 6^; and (2) suburban pixels in a city falling into the urban area of a neighboring city^5^. The Spearman’s rank correlation coefficient, a non-parametric method, was applied to quantity the correlation between SUHII and the ΔLMs. The Spearman’s rank correlation coefficient ranges from −1 to 1, and a higher absolute value indicates a more powerful correlation. A positive correlation coefficient means a positive correlation between SUHII and the ΔLMs, and vice versa. The significance test was performed by a two-tailed t-test, and the standard 0.05 significance level was adopted in the analysis.

### Data availability

All the land surface temperature data can be downloaded on the MODIS product website (https://lpdaac.usgs.gov/data_access/data_pool). The land-cover classification datasets are provided by the Chinese Academy of Sciences. Other relevant data in this study are available from the authors.

## Electronic supplementary material

Supplementary information
