# Supplementary material for: Assessing the relationship between surface urban heat islands and landscape patterns across climatic zones in China
Source: Sci Rep. 2017 Aug 24;7:9337. doi: 10.1038/s41598-017-09628-w (PMC5571207; doi:10.1038/s41598-017-09628-w)
Supplement: Supplementary file 1 — Supplementary information [file 41598_2017_9628_MOESM1_ESM.pdf]

Supplementary information

Assessing the relationship between surface urban heat islands and landscape patterns across climatic zones in China

Qiquan Yang[2], Xin Huang[1, 2, *], Jiayi Li[1, *]

[1] School of Remote Sensing and Information Engineering, Wuhan University, 129 Luoyu Road, Wuhan 430079, China

[2] State Key Laboratory of Information Engineering in Surveying, Mapping and Remote Sensing, Wuhan University, Wuhan 430079, China

[*] Corresponding authors.

E-mail address: xhuang@whu.edu.cn (X. Huang), zjjerica@whu.edu.cn (J. Li)

Table S1 Urban−suburban difference (mean ± SD) in landscape metrics at both landscape level and class level across climatic zones and China. See Table 1 and Fig. 1 for details of the landscape metrics (PLAND, SHDI, PD, MSI, CI, and CONTAG) and climatic zones (EW, W, A, S, and TS), respectively. Δ means the difference between urban and suburban.

		China	EW	W	A	S	TS
Landscape level							
ΔPD		−0.37 ± 0.33	−0.30 ± 0.34	−0.43 ± 0.32	−0.25 ± 0.21	−0.44 ± 0.28	−0.46 ± 0.46
ΔMSI		0.27 ± 1.66	0.53 ± 1.81	0.27 ± 1.51	0.24 ± 1.09	−0.3 ± 1.95	0.25 ± 0.67
ΔCONTAG		−5.53 ± 6.47	−5.19 ± 5.83	−5.26 ± 6.48	−7.38 ± 7.76	−5.08 ± 7.27	−8.00 ± 4.67
ΔSHDI		0.16 ± 0.17	0.16 ± 0.16	0.14 ± 0.18	0.21 ± 0.20	0.14 ± 0.17	0.23 ± 0.14
Class level							
ΔPLAND	Bare land	−1.58 ± 5.17	0.10 ± 0.39	−0.16 ± 1.22	−8.84 ± 10.22	−0.52 ± 1.53	−1.07 ± 2.22
	Built-up	29.64 ± 8.59	28.72 ± 9.04	30.50 ± 7.75	31.86 ± 7.44	31.56 ± 8.19	19.00 ± 6.12
	Waterbody	−0.01 ± 0.04	−0.01 ± 0.05	0.00 ± 0.04	−0.01 ± 0.03	0.00 ± 0.01	−0.02 ± 0.05
	Wetland	−0.53 ± 2.55	−0.77 ± 2.28	−0.29 ± 1.74	−0.49 ± 1.24	−0.50 ± 3.43	0.01 ± 5.37
	Cultivated	−15.00 ± 14.06	−13.47 ± 13.6	−18.03 ± 15.29	−13.18 ± 12.82	−16.90 ± 10.90	2.54 ± 8.61
	Grassland	−4.66 ± 7.71	−1.58 ± 3.06	−4.60 ± 7.38	−10.55 ± 11.92	−5.09 ± 7.36	−13.98 ± 9.97
	Forest	−9.71 ± 11.15	−13.28 ± 11.81	−8.92 ± 11.24	−0.63 ± 2.65	−8.95 ± 9.35	−7.03 ± 10.01
ΔPD	Bare land	−0.01 ± 0.04	0.00 ± 0.01	0.00 ± 0.01	−0.06 ± 0.08	−0.01 ± 0.02	−0.05 ± 0.06
	Built-up	−0.16 ± 0.19	−0.18 ± 0.19	−0.17 ± 0.20	−0.09 ± 0.12	−0.15 ± 0.16	0.00± 0.10
	Waterbody	−0.02 ± 0.14	−0.03 ± 0.18	−0.02 ± 0.13	0.00 ± 0.04	−0.01 ± 0.04	−0.01 ± 0.18
	Wetland	−0.01 ± 0.03	−0.01 ± 0.02	0.00 ± 0.02	−0.01 ± 0.02	−0.01 ± 0.03	−0.05 ± 0.08
	Cultivated	−0.05 ± 0.15	−0.02 ± 0.11	−0.08 ± 0.20	−0.02 ± 0.07	−0.10 ± 0.10	−0.07 ± 0.14
	Grassland	−0.06 ± 0.11	−0.03 ± 0.06	−0.08 ± 0.14	−0.06 ± 0.07	−0.06 ± 0.09	−0.18 ± 0.18
	Forest	−0.07 ± 0.12	−0.04 ± 0.12	−0.09 ± 0.12	−0.02 ± 0.06	−0.10 ± 0.12	−0.18 ± 0.15
ΔMSI	Bare land	0.03 ± 1.05	0.19 ± 1.18	−0.20 ± 1.05	0.31 ± 0.75	−0.13 ± 1.00	0.09 ± 1.14
	Built-up	0.15 ± 0.22	0.14 ± 0.13	0.13 ± 0.14	0.18 ± 0.27	0.13 ± 0.13	0.49 ± 0.71
	Waterbody	−0.04 ± 0.35	−0.01 ± 0.18	−0.06 ± 0.4	0.00 ± 0.55	−0.06 ± 0.42	−0.14 ± 0.42
	Wetland	0.10 ± 0.92	0.02 ± 0.81	0.13 ± 1.05	−0.17 ± 0.70	0.31 ± 1.01	0.36 ± 0.83
	Cultivated	0.13 ± 0.46	0.00 ± 0.26	0.22 ± 0.55	0.11 ± 0.56	0.23 ± 0.52	0.22 ± 0.21
	Grassland	−0.02 ± 0.53	−0.03 ± 0.5	0.04 ± 0.55	−0.05 ± 0.56	−0.11 ± 0.54	0.13 ± 0.39
	Forest	0.00 ± 0.48	−0.05 ± 0.44	0.00 ± 0.36	0.28 ± 0.76	−0.05 ± 0.45	−0.01 ± 0.80
ΔCI	Bare land	−0.05 ± 0.56	0.04 ± 0.7	−0.14 ± 0.58	−0.03 ± 0.21	−0.06 ± 0.55	−0.07 ± 0.51
	Built-up	0.06 ± 0.10	0.05 ± 0.03	0.04 ± 0.02	0.08 ± 0.17	0.05 ± 0.02	0.25 ± 0.38
	Waterbody	−0.02 ± 0.27	−0.01 ± 0.17	−0.02 ± 0.29	−0.04 ± 0.36	0.00 ± 0.36	−0.12 ± 0.25
	Wetland	−0.03 ± 0.34	−0.03 ± 0.3	−0.01 ± 0.44	−0.11 ± 0.32	−0.03 ± 0.28	−0.06 ± 0.29
	Cultivated	0.02 ± 0.06	0.02 ± 0.03	0.04 ± 0.04	−0.02 ± 0.17	0.03 ± 0.02	0.01 ± 0.02
	Grassland	0.00 ± 0.25	0.02 ± 0.25	0.03 ± 0.24	−0.08 ± 0.29	−0.05 ± 0.25	0.03 ± 0.02
	Forest	0.00 ± 0.19	0.00 ± 0.15	−0.01 ± 0.14	0.10 ± 0.28	−0.04 ± 0.22	−0.14 ± 0.36

Table S2 Landscape metrics (mean ± SD) in the urban areas at both landscape level and class level across climatic zones and China. See Table 1 and Fig. 1 for details of the landscape metrics (PLAND, SHDI, PD, MSI, CI, and CONTAG) and climatic zones (EW, W, A, S, and TS), respectively.

		China	EW	W	A	S	TS
Landscape level							
PD		0.89 ± 0.49	1.15 ± 0.48	0.83 ± 0.44	0.56 ± 0.43	0.68 ± 0.44	0.80 ± 0.40
MSI		1.69 ± 0.16	1.67 ± 0.15	1.67 ± 0.17	1.74 ± 0.13	1.78 ± 0.14	1.69 ± 0.16
CONTAG		64.00 ± 7.14	63.62 ± 6.45	64.58 ± 8.38	64.85 ± 4.18	64.11 ± 6.7	60.72 ± 8.82
SHDI		1.06 ± 0.20	1.07 ± 0.17	1.00 ± 0.21	1.11 ± 0.18	1.09 ± 0.20	1.14 ± 0.31
Class level							
PLAND	Bare land	3.76 ± 10.90	0.36 ± 0.72	0.50 ± 0.82	14.11 ± 17.83	0.72 ± 1.79	10.34 ± 21.49
	Built-up	38.85 ± 11.01	39.26 ± 8.94	40.9 ± 11.77	37.72 ± 8.88	39.44 ± 11.32	20.83 ± 7.26
	Waterbody	0.04 ± 0.05	0.05 ± 0.06	0.03 ± 0.05	0.02 ± 0.02	0.02 ± 0.02	0.04 ± 0.04
	Wetland	1.88 ± 2.66	1.41 ± 2.05	1.37 ± 1.98	1.38 ± 1.59	3.05 ± 3.77	4.88 ± 3.55
	Cultivated	38.62 ± 14.27	37.29 ± 13.14	43.29 ± 12.87	32.93 ± 17.46	37.2 ± 14.36	29.7 ± 17.57
	Grassland	8.58 ± 13.38	3.06 ± 4.11	7.32 ± 9.39	18.48 ± 18.2	8.92 ± 11.84	36.41 ± 25.12
	Forest	13.57 ± 15.27	19.95 ± 15.93	9.56 ± 11.72	1.86 ± 1.93	13.15 ± 16.29	15.24 ± 21.69
PD	Bare land	0.02 ± 0.02	0.01 ± 0.01	0.01 ± 0.02	0.04 ± 0.03	0.02 ± 0.02	0.04 ± 0.03
	Built-up	0.31 ± 0.20	0.34 ± 0.2	0.35 ± 0.18	0.24 ± 0.32	0.24 ± 0.13	0.19 ± 0.20
	Waterbody	0.12 ± 0.18	0.19 ± 0.22	0.10 ± 0.17	0.06 ± 0.07	0.04 ± 0.03	0.16 ± 0.14
	Wetland	0.03 ± 0.03	0.03 ± 0.03	0.02 ± 0.02	0.03 ± 0.02	0.03 ± 0.03	0.07 ± 0.07
	Cultivated	0.2 ± 0.18	0.28 ± 0.19	0.17 ± 0.17	0.09 ± 0.06	0.15 ± 0.16	0.21 ± 0.10
	Grassland	0.10 ± 0.13	0.08 ± 0.10	0.11 ± 0.15	0.09 ± 0.07	0.12 ± 0.18	0.19 ± 0.14
	Forest	0.17 ± 0.18	0.26 ± 0.22	0.13 ± 0.13	0.05 ± 0.05	0.13 ± 0.10	0.15 ± 0.14
MSI	Bare land	1.76 ± 0.47	1.67 ± 0.38	1.77 ± 0.36	2.05 ± 0.66	1.58 ± 0.26	1.91 ± 0.66
	Built-up	1.61 ± 0.17	1.66 ± 0.18	1.56 ± 0.18	1.61 ± 0.14	1.58 ± 0.11	1.60 ± 0.20
	Waterbody	1.10 ± 0.08	1.10 ± 0.07	1.11 ± 0.09	1.10 ± 0.08	1.11 ± 0.07	1.08 ± 0.08
	Wetland	2.14 ± 0.73	1.96 ± 0.5	2.16 ± 0.69	2.12 ± 0.76	2.46 ± 1.01	2.32 ± 0.76
	Cultivated	2.25 ± 0.46	2.1 ± 0.28	2.39 ± 0.57	2.26 ± 0.45	2.37 ± 0.46	1.96 ± 0.17
	Grassland	1.83 ± 0.31	1.66 ± 0.21	1.92 ± 0.32	1.97 ± 0.32	1.91 ± 0.34	1.89 ± 0.26
	Forest	1.74 ± 0.27	1.73 ± 0.17	1.71 ± 0.27	1.78 ± 0.38	1.79 ± 0.33	1.87 ± 0.51
CI	Bare land	0.94 ± 0.04	0.93 ± 0.04	0.93 ± 0.04	0.96 ± 0.03	0.94 ± 0.04	0.96 ± 0.01
	Built-up	0.95 ± 0.02	0.94 ± 0.02	0.95 ± 0.01	0.96 ± 0.02	0.96 ± 0.01	0.96 ± 0.02
	Waterbody	0.46 ± 0.16	0.43 ± 0.11	0.46 ± 0.21	0.47 ± 0.16	0.49 ± 0.14	0.47 ± 0.15
	Wetland	0.92 ± 0.04	0.92 ± 0.04	0.93 ± 0.04	0.93 ± 0.03	0.93 ± 0.03	0.94 ± 0.02
	Cultivated	0.92 ± 0.03	0.91 ± 0.02	0.92 ± 0.02	0.94 ± 0.02	0.93 ± 0.03	0.94 ± 0.03
	Grassland	0.93 ± 0.03	0.93 ± 0.03	0.92 ± 0.03	0.95 ± 0.02	0.93 ± 0.04	0.94 ± 0.03
	Forest	0.93 ± 0.03	0.93 ± 0.02	0.93 ± 0.03	0.93 ± 0.03	0.93 ± 0.03	0.94 ± 0.02

Table S3 Spearman's rank correlation coefficients between the surface urban heat island intensity (SUHII) and the urban−suburban difference in the landscape metrics (ΔLMs) of bare land across climatic zones and China. See Table 1 and Fig. 1 for details of the landscape metrics (PLAND, PD, MSI, and CI) and climatic zones (EW, W, A, S, and TS), respectively. Δ means the difference between urban and suburban.

Landscape metrics of bare land	Climatic zone	Annual day	Annual night	Summer day	Summer night	Winter day	Winter night
ΔPLAND	China	0.14	−0.18 [c]	0.19 [c]	−0.18 [c]	0.06	−0.20 [c]
	EW	0.08	−0.11	−0.04	−0.24	−0.29	0.05
	W	−0.05	−0.12	0.11	−0.08	−0.02	−0.13
	A	0.39	0.15	0.48 [c]	0.20	0.01	−0.03
	S	−0.08	−0.06	0.05	−0.04	−0.22	−0.10
	TS	−0.08	−0.22	0.19	−0.15	−0.09	−0.24
ΔPD	China	0.12	−0.11	0.17 [c]	−0.11	0.06	−0.14
	EW	−0.13	−0.09	−0.09	−0.20	−0.25	−0.01
	W	0.04	0.06	0.17	0.13	0.09	0.06
	A	0.22	0.24	0.29	0.23	−0.09	0.06
	S	−0.08	−0.20	0.10	−0.14	−0.22	−0.28
	TS	0.29	−0.19	0.23	−0.04	0.31	−0.05
ΔMSI	China	−0.16 [c]	−0.09	−0.13	−0.11	−0.05	−0.05
	EW	0.05	−0.10	−0.11	−0.10	−0.10	0.00
	W	−0.04	−0.20	0.09	−0.25	0.14	−0.14
	A	−0.47 [c]	−0.14	−0.43 [c]	−0.22	−0.27	−0.02
	S	−0.21	−0.02	−0.19	−0.10	−0.20	0.01
	TS	−0.43	0.49	−0.20	0.36	−0.38	0.59
ΔCI	China	0.00	−0.08	0.04	−0.13	0.04	−0.03
	EW	0.03	−0.05	−0.10	−0.13	−0.21	0.05
	W	−0.09	−0.27	−0.02	−0.31	0.17	−0.21
	A	−0.20	−0.06	−0.21	−0.23	0.00	0.06
	S	0.06	0.17	0.21	0.09	−0.10	0.21
	TS	0.25	0.25	0.34	0.48	0.15	0.15

[a] significant at the 0.001 level; [b] significant at the 0.01 level; [c] significant at the 0.05 level.

Table S4 Spearman's rank correlation coefficients between the surface urban heat island intensity (SUHII) and the urban−suburban difference in the landscape metrics (ΔLMs) of waterbody across climatic zones and China. See Table 1 and Fig. 1 for details of the landscape metrics (PLAND, PD, MSI, and CI) and climatic zones (EW, W, A, S, and TS), respectively. Δ means the difference between urban and suburban.

Landscape metrics of waterbody	Climatic zone	Annual day	Annual night	Summer day	Summer night	Winter day	Winter night
ΔPLAND	China	−0.04	0.13 [c]	−0.07	0.13 [c]	−0.06	0.10
	EW	0.06	0.17	0.01	0.16	0.00	0.03
	W	−0.07	0.08	−0.14	0.09	−0.12	0.11
	A	−0.63 [a]	0.28	−0.60 [b]	0.11	−0.37	0.27
	S	0.00	−0.07	−0.08	−0.04	0.15	−0.07
	TS	0.43	0.88 [c]	0.14	0.64	−0.02	0.93 [b]
ΔPD	China	−0.08	0.12	−0.08	0.12 [c]	−0.07	0.10
	EW	0.02	0.18	−0.02	0.19 [c]	0.05	0.04
	W	−0.08	0.12	−0.15	0.09	−0.15	0.15
	A	−0.34	0.24	−0.26	0.02	−0.33	0.39 [c]
	S	−0.21	−0.27	−0.11	−0.21	0.00	−0.32 [c]
	TS	0.50	0.76	0.10	0.50	0.05	0.79 [c]
ΔMSI	China	−0.03	−0.04	−0.01	−0.04	−0.04	−0.03
	EW	−0.02	−0.06	0.02	−0.07	−0.13	−0.05
	W	−0.08	0.08	−0.07	−0.02	−0.10	0.11
	A	−0.21	−0.07	−0.16	−0.12	−0.07	0.01
	S	0.07	−0.07	0.05	−0.01	0.08	−0.10
	TS	0.21	0.00	0.67	0.10	0.05	−0.12
ΔCI	China	0.02	0.00	0.01	0.03	0.02	0.01
	EW	−0.01	−0.04	0.05	−0.02	−0.11	0.05
	W	0.00	0.02	−0.05	0.02	0.03	0.02
	A	−0.29	−0.21	−0.16	−0.31	−0.13	−0.11
	S	0.21	0.12	0.08	0.12	0.30 [c]	0.10
	TS	−0.26	0.07	0.33	0.26	−0.45	0.02

[a] significant at the 0.001 level; [b] significant at the 0.01 level; [c] significant at the 0.05 level.

Table S5 Spearman's rank correlation coefficients between the surface urban heat island intensity (SUHII) and the urban−suburban difference in the landscape metrics (ΔLMs) of wetland across climatic zones and China. See Table 1 and Fig. 1 for details of the landscape metrics (PLAND, PD, MSI, and CI) and climatic zones (EW, W, A, S, and TS), respectively. Δ means the difference between urban and suburban.

Landscape metrics of wetland	Climatic zone	Annual day	Annual night	Summer day	Summer night	Winter day	Winter night
ΔPLAND	China	−0.14 [c]	0.08	−0.15 [c]	0.12 [c]	−0.06	0.05
	EW	−0.08	0.22 [c]	−0.16	0.26 [b]	0.12	0.17
	W	−0.23	0.01	−0.26 [c]	0.04	−0.06	−0.01
	A	−0.40	0.16	−0.34	−0.04	−0.33	0.23
	S	−0.25	−0.25	−0.25	−0.12	−0.11	−0.33 [c]
	TS	0.52	0.20	0.43	0.23	0.10	0.06
ΔPD	China	0.07	0.12 [c]	0.01	0.13 [c]	0.11	0.10
	EW	−0.15	0.13	−0.18	0.10	0.04	0.12
	W	0.08	−0.07	0.09	0.06	0.11	−0.16
	A	−0.01	0.18	−0.50 [c]	0.06	0.08	0.23
	S	0.07	0.18	0.00	0.10	0.11	0.20
	TS	0.83 [b]	0.71 [c]	0.77 [b]	0.65 [c]	0.26	0.57
ΔMSI	China	0.00	0.05	−0.05	0.05	0.01	−0.01
	EW	0.17	−0.02	−0.03	−0.03	0.18	−0.08
	W	−0.07	0.12	−0.06	0.14	−0.02	0.09
	A	−0.04	0.03	−0.21	0.14	0.08	−0.19
	S	−0.10	0.08	0.02	0.07	−0.09	−0.04
	TS	0.24	−0.20	0.31	−0.04	0.24	−0.37
ΔCI	China	−0.11	0.06	−0.10	0.06	−0.03	0.05
	EW	−0.12	0.10	−0.04	0.06	0.01	0.06
	W	−0.08	0.16	−0.19	0.18	−0.05	0.16
	A	−0.19	−0.38	−0.04	−0.34	0.05	−0.23
	S	−0.16	0.03	−0.19	0.16	−0.17	−0.09
	TS	0.74 [b]	−0.20	0.56	−0.17	0.75 [b]	−0.26

[a] significant at the 0.001 level; [b] significant at the 0.01 level; [c] significant at the 0.05 level.

Table S6 Spearman's rank correlation coefficients between the surface urban heat island intensity (SUHII) and the urban−suburban difference in landscape configurational metrics of forest across climatic zones and China. See Table 1 and Fig. 1 for details of the landscape metrics (PD, MSI, and CI) and climatic zones (EW, W, A, S, and TS), respectively. Δ means the difference between urban and suburban.

Landscape configurational metrics of forest	Climatic zone	Annual day	Annual night	Summer day	Summer night	Winter day	Winter night
ΔPD	China	−0.12 [c]	0.07	−0.10	−0.05	−0.11 [c]	0.07
	EW	0.02	0.06	0.04	−0.03	−0.07	−0.06
	W	−0.13	0.26 [c]	−0.18	0.02	−0.33 [a]	0.36 [a]
	A	−0.33	0.14	−0.52 [b]	0.03	−0.22	0.17
	S	−0.13	0.10	−0.07	−0.16	−0.03	0.12
	TS	−0.44	0.19	−0.34	0.20	−0.42	0.27
ΔMSI	China	−0.01	0.02	0.00	0.07	0.03	−0.02
	EW	0.13	−0.06	0.08	−0.03	0.19 [c]	−0.14
	W	0.02	0.04	−0.07	0.18	0.08	−0.01
	A	−0.26	−0.12	−0.27	−0.23	−0.16	−0.05
	S	−0.09	−0.19	0.05	−0.12	0.00	−0.21
	TS	0.19	0.73 [b]	0.54	0.68 [c]	−0.26	0.57 [c]
ΔCI	China	−0.02	−0.06	−0.01	−0.01	0.00	−0.08
	EW	−0.13	0.06	−0.08	0.14	0.05	0.01
	W	−0.05	−0.13	−0.10	−0.11	−0.07	−0.11
	A	0.35	−0.01	0.13	0.07	0.27	−0.20
	S	−0.11	−0.17	0.02	−0.07	−0.12	−0.20
	TS	0.08	0.40	0.31	0.40	−0.11	0.19

[a] significant at the 0.001 level; [b] significant at the 0.01 level; [c] significant at the 0.05 level.

Table S7 Spearman's rank correlation coefficients between the surface urban heat island intensity (SUHII) and the urban−suburban difference in landscape configurational metrics of grassland across climatic zones and China. See Table 1 and Fig. 1 for details of the landscape metrics (PD, MSI, and CI) and climatic zones (EW, W, A, S, and TS), respectively. Δ means the difference between urban and suburban.

Landscape configurational metrics of grassland	Climatic zone	Annual day	Annual night	Summer day	Summer night	Winter day	Winter night
ΔPD	China	0.03	−0.03	0.08	−0.16 [b]	−0.08	0.06
	EW	−0.13	−0.17	−0.05	−0.34 [a]	−0.41 [a]	−0.02
	W	0.00	0.13	0.02	−0.08	−0.14	0.28 [c]
	A	−0.03	0.20	0.04	0.19	−0.09	0.08
	S	0.27 [c]	0.10	0.29 [c]	0.01	0.13	0.10
	TS	−0.65 [c]	0.16	−0.52 [c]	0.14	−0.55 [c]	0.19
ΔMSI	China	−0.02	−0.01	−0.07	0.03	0.03	−0.02
	EW	−0.05	0.05	−0.01	−0.03	−0.08	0.00
	W	0.02	0.01	−0.06	0.13	0.09	−0.01
	A	0.29	−0.25	−0.13	−0.13	0.59 [a]	−0.34
	S	−0.19	−0.06	−0.14	−0.03	−0.14	−0.10
	TS	0.35	0.12	0.25	0.00	0.27	0.24
ΔCI	China	−0.08	0.06	−0.10	0.06	−0.03	0.08
	EW	0.04	0.09	−0.02	0.14	0.04	0.06
	W	−0.08	0.16	−0.08	0.11	−0.16	0.16
	A	−0.01	0.04	−0.24	0.05	0.22	0.13
	S	−0.07	0.11	−0.10	0.14	−0.03	0.09
	TS	0.35	−0.21	0.16	−0.13	0.45	−0.19

[a] significant at the 0.001 level; [b] significant at the 0.01 level; [c] significant at the 0.05 level.

Table S8 Spearman's rank correlation coefficients between the surface urban heat island intensity (SUHII) and the urban−suburban difference in landscape configurational metrics of cultivated land across climatic zones and China. See Table 1 and Fig. 1 for details of the landscape metrics (PD, MSI, and CI) and climatic zones (EW, W, A, S, and TS), respectively. Δ means the difference between urban and suburban.

Landscape configurational metrics of cultivated land	Climatic zone	Annual day	Annual night	Summer day	Summer night	Winter day	Winter night
ΔPD	China	0.01	0.09	0.02	−0.05	−0.05	0.12 [c]
	EW	−0.07	0.04	0.02	−0.10	−0.38 [a]	0.11
	W	0.00	0.24 [c]	−0.05	0.03	−0.14	0.33 [a]
	A	0.08	−0.06	0.11	−0.04	0.07	−0.09
	S	0.23	0.24	0.08	−0.07	0.13	0.36 [b]
	TS	0.22	0.70 [c]	0.52	0.59	−0.15	0.66 [c]
ΔMSI	China	0.07	0.08	0.04	0.08	−0.08	0.04
	EW	0.11	−0.18 [c]	0.01	−0.06	0.18	−0.26 [b]
	W	−0.07	0.00	−0.01	−0.02	−0.16	−0.04
	A	−0.04	−0.08	0.00	−0.13	0.00	−0.03
	S	0.20	−0.01	0.27 [c]	−0.04	0.08	0.02
	TS	−0.03	0.14	−0.09	0.05	0.14	0.25
ΔCI	China	−0.03	0.12 [c]	0.03	−0.03	−0.34 [a]	0.21 [a]
	EW	−0.13	−0.13	0.01	−0.29 [b]	−0.48 [a]	0.10
	W	−0.23 [c]	0.20 [c]	−0.27 [b]	−0.06	−0.47 [a]	0.33 [a]
	A	0.32	0.35	0.63 [a]	0.33	−0.06	0.26
	S	0.15	0.25	0.17	0.14	−0.06	0.31 [c]
	TS	−0.53	−0.51	−0.65 [c]	−0.45	−0.08	−0.64 [c]

[a] significant at the 0.001 level; [b] significant at the 0.01 level; [c] significant at the 0.05 level.
